# *In vitro* and *in vivo* Effect of Exogenous Farnesol Exposure Against *Candida auris*

**DOI:** 10.3389/fmicb.2020.00957

**Published:** 2020-05-20

**Authors:** Fruzsina Nagy, Eszter Vitális, Ágnes Jakab, Andrew M. Borman, Lajos Forgács, Zoltán Tóth, László Majoros, Renátó Kovács

**Affiliations:** ^1^Department of Medical Microbiology, Faculty of Medicine, University of Debrecen, Debrecen, Hungary; ^2^Doctoral School of Pharmaceutical Sciences, University of Debrecen, Debrecen, Hungary; ^3^Hospital Hygiene Ward, Clinical Centre, University of Debrecen, Debrecen, Hungary; ^4^Department of Molecular Biotechnology and Microbiology, Faculty of Science and Technology, Institute of Biotechnology, University of Debrecen, Debrecen, Hungary; ^5^UK National Mycology Reference Laboratory, Public Health England, Bristol, United Kingdom; ^6^Faculty of Pharmacy, University of Debrecen, Debrecen, Hungary

**Keywords:** biofilm, oxidative stress, virulence, *in vivo*, synergy, triazoles, quorum-sensing, therapy

## Abstract

The spreading of multidrug-resistant *Candida auris* is considered as an emerging global health threat. The number of effective therapeutic regimens is strongly limited; therefore, development of novel strategies is needed. Farnesol is a quorum-sensing molecule with a potential antifungal and/or adjuvant effect; it may be a promising candidate in alternative treatment against *Candida* species including *C. auris*. To examine the effect of farnesol on *C. auris*, we performed experiments focusing on growth, biofilm production ability, production of enzymes related to oxidative stress, triazole susceptibility and virulence. Concentrations ranging from 100 to 300 μM farnesol caused a significant growth inhibition against *C. auris* planktonic cells for 24 h (*p* < 0.01–0.05). Farnesol treatment showed a concentration dependent inhibition in terms of biofilm forming ability of *C. auris*; however, it did not inhibit significantly the biofilm development at 24 h. Nevertheless, the metabolic activity of adhered farnesol pre-exposed cells (75 μM) was significantly diminished at 24 h depending on farnesol treatment during biofilm formation (*p* < 0.001–0.05). Moreover, 300 μM farnesol exerted a marked decrease in metabolic activity against one-day-old biofilms between 2 and 24 h (*p* < 0.001). Farnesol increased the production of reactive species remarkably, as revealed by 2′,7′-dichlorofluorescein (DCF) assay {3.96 ± 0.89 [nmol DCF (OD_640_)^–1^] and 23.54 ± 4.51 [nmol DCF (OD_640_)^–1^] for untreated cells and farnesol exposed cells, respectively; *p* < 0.001}. This was in line with increased superoxide dismutase level {85.69 ± 5.42 [munit (mg protein)^–1^] and 170.11 ± 17.37 [munit (mg protein)^–1^] for untreated cells and farnesol exposed cells, respectively; *p* < 0.001}, but the catalase level remained statistically comparable between treated and untreated cells (*p* > 0.05). Concerning virulence-related enzymes, exposure to 75 μM farnesol did not influence phospholipase or aspartic proteinase activity (*p* > 0.05). The interaction between fluconazole, itraconazole, voriconazole, posaconazole, isavuconazole and farnesol showed clear synergism (FICI ranges from 0.038 to 0.375) against one-day-old biofilms. Regarding *in vivo* experiments, daily 75 μM farnesol treatment decreased the fungal burden in an immunocompromised murine model of disseminated candidiasis, especially in case of inocula pre-exposed to farnesol (*p* < 0.01). In summary, farnesol shows a promising therapeutic or adjuvant potential in traditional or alternative therapies such as catheter lock therapy.

## Introduction

*Candida auris* is an emerging fungal pathogen causing outbreaks in healthcare settings with unacceptably high mortality rates ranging from 28 to 78% depending on the country (Jeffery-Smith et al., 2017; Eyre et al., 2018). To date, 39 countries have reported *C. auris* associated infections (Jeffery-Smith et al., 2017; Eyre et al., 2018; Kean et al., 2020). Based on last published data, the number of confirmed *C. auris* infections were 620 and 988 in Europe and United States of America, respectively (European Centre for Disease Prevention and Control, 2018; Centers for Disease Control and Prevention, 2019). Nosocomial *C. auris* outbreaks were reported from several countries including India, South Africa, Venezuela, Pakistan, and the United States (Lockhart et al., 2017; Vallabhaneni et al., 2017; Belkin et al., 2018). Previously, genetic analyses revealed more genetically unrelated clonal populations across three different continents. These clades are commonly classified as South African, South Asian, East Asian, and South American clades (Lockhart et al., 2017). In addition, a recent study described a fifth *C. auris* clade in Iran from patient who never traveled outside that country (Abastabar et al., 2019; Chow et al., 2019).

Over 90% of clinical isolates are resistant to fluconazole whereas resistance to newer triazoles is variable (Dudiuk et al., 2019; Romera et al., 2019). The ratio of strains resistant to amphotericin B ranges from 8 to 50%, while echinocandin resistance remains infrequent (2 to 8%) (Dudiuk et al., 2019). Alarmingly, isolates of *C. auris* with resistance to all three major antifungal classes have been reported in multiple countries including the United States (Ostrowsky et al., 2020). These multidrug-resistant strains may remain susceptible to nystatin and terbinafine (Sarma and Upadhyay, 2017). *C. auris* biology have been extensively covered in recent papers (Rossato and Colombo, 2018; Casadevall et al., 2019), however, the data about potential alternative treatment strategies remain scarce (Wall et al., 2018); therefore, there is an urgent need for the development of new antifungal therapies. In addition, multidrug-resistance is significantly more frequently reported in the case of *C. auris* biofilms (Kean and Ramage, 2019). Thus, although the capacity to form biofilms is strain dependent in *C. auris*, they frequently pose a remarkable therapeutic challenge, especially because *C. auris* biofilms also have a considerable virulence capacity (Kean and Ramage, 2019). Since data collected with *Candida albicans* biofilms cannot be extrapolated to *C. auris* directly, such studies are urgently needed to meet this novel challenge (Kean and Ramage, 2019).

Farnesol is a fungal quorum-sensing molecule that inhibits yeast-to-hyphae transition and promotes reverse morphogenesis in *C. albicans* (Hornby et al., 2001). Based on recent studies, farnesol acts synergistically with several antifungal agents against *C. albicans, Candida glabrata*, *Candida tropicalis* as well as against *Candida parapsilosis* planktonic cells and/or biofilms (Katragkou et al., 2015; Kovács et al., 2016; Monteiro et al., 2017; Agustín et al., 2019), thus it has been proposed as a potential adjuvant therapeutic agent. In addition, its therapeutic potential has already been confirmed against *C. albicans* in murine models of mucosal infection (Hisajima et al., 2008; Bozó et al., 2016). Although farnesol is not beneficial in systemic infections caused by *C. albicans* (Navarathna et al., 2007), those data cannot necessarily be extrapolated to non-*albicans* species including *C. auris* (Semreen et al., 2019).

This study examines the effect of farnesol exposure on growth, biofilm production, oxidative stress-related enzyme production, triazole susceptibility and virulence of *C. auris*, in order to explore the background of the previously observed antifungal effect.

## Materials and Methods

### Organisms

Three *C. auris* isolates (isolates 10, 12, and 27) obtained from National Mycology Reference Laboratory, United Kingdom were used together with the SC5314 *C. albicans* reference strain. All three *C. auris* strains derived from the South Asian/Indian lineage (Borman et al., 2017). All *C. auris* isolates tested showed non-aggregating phenotype, which exhibit comparable pathogenicity to that of *C. albicans* (Borman et al., 2016).

### Toxicity Experiments

Ten μM, 50 μM, 150 μM, and 300 μM farnesol were evaluated in terms of toxicity to the Caco-2 cell line using a 3-(4,5-dimethyl-2-thiazolyl)-2,5-diphenyl-2H-tetrazolium bromide (MTT) assay (Sigma, Budapest, Hungary) (Berridge et al., 2005). No toxicity was observed with any concentration of farnesol.

### Growth Related Experiments for Planktonic Cells

The effect of pre-exposure and continuous farnesol treatment on *C. auris* and *C. albicans* planktonic cells was tested in RPMI-1640 (with L-glutamine and without bicarbonate, pH 7.0 with MOPS; Sigma, Budapest, Hungary) in two experimental settings: (i) effect of various farnesol concentrations against planktonic cells, (ii) effect of various farnesol concentrations against planktonic cells pre-exposed with farnesol (75 μM) for 24-h. Seventy-five μM farnesol was chosen as pre-exposure concentration because it corresponds to approximately double the amount of physiological farnesol production of *C. albicans* (Weber et al., 2008).

Farnesol was obtained as 3M stock solution, which was diluted to a 30 mM working stock solution in 100% methanol. The working concentrations of farnesol were prepared in RPMI-1640 medium. Drug-free control was supplemented with 1% (vol/vol) methanol (Bozó et al., 2016; Kovács et al., 2016; Nagy et al., 2019). Farnesol concentrations tested were 10, 50, 100, and 300 μM in all experiments.

Living cell number of planktonic cells was determined using time-kill experiments (Kovács et al., 2014, 2017). Briefly, samples (100 μL) were removed at 0, 2, 4, 6, 8, 10, 12, and 24 h, serially diluted tenfold, plated (4 × 30 μL) onto Sabouraud dextrose agar and incubated at 35°C for 48 h. All isolates were tested in three independent experiments and the mean of the three values was used in the analysis. At given time points, one-way ANOVA with Dunnett’s post-testing was used to analyze the effect on living cell number exerted by different farnesol concentrations compared to untreated control.

### Evaluation of Extracellular Phospholipase and Aspartic Proteinase Activities Exerted by Farnesol Exposure

Extracellular phospholipase production by farnesol-exposed (75 μM) and untreated *C. auris* and *C. albicans* cells was examined on egg yolk medium [5.85% (wt/vol) NaCl, 0.05% (wt/vol) CaCl_2_, and 10% (vol/vol) sterile egg yolk (Sigma, Budapest, Hungary)]. Aspartic proteinase activity was evaluated on solid medium supplemented with bovine serum albumin [0.02% (wt/vol) MgSO_4_ × 7H_2_O, 0.25% (wt/vol) K_2_HPO_4_, 0.5% (wt/vol) NaCl, 0.1% (wt/vol) yeast extract, 2% (wt/vol) glucose and 0.25% (wt/vol) bovine serum albumin (Sigma, Budapest, Hungary) agar medium]. In case of both assay, 5 μL suspensions of 1 × 10^7^ cells/mL were inoculated onto agar plates as described previously (Kantarcioglu and Yücel, 2002). Colony diameters and precipitation zones (Pz) were measured after 7 days of incubation at 35°C (Price et al., 1982). Enzyme activities were measured in three independent experiments for each isolate and are presented as means ± standard deviations. Statistical analysis of reactive species and enzyme production data were performed by paired Student’s *t-*test using GraphPad Prism 6.05 software. The differences between values for treated and control cells were considered significant if the *p*-value was <0.05.

### Reactive Species Production and Antioxidant Enzyme Activities Exerted by Farnesol Exposure

Reactive species were measured in the presence or absence of 1-day farnesol (75 μM) exposure in RPMI-1640 by a reaction that converts 2′,7′-dichlorofluorescin diacetate to 2′,7′-dichlorofluorescein (DCF) (Sigma, Budapest, Hungary) (Jakab et al., 2015, 2019). The amount of DCF produced is proportional to the quantity of reactive species. Catalase and superoxide dismutase activities were determined as described previously by Jakab et al. (2015, 2019). Reactive species and enzyme activities were measured in three independent experiments for each isolate and are presented as means ± standard deviations. Statistical comparisons of reactive species and enzyme production data were performed by paired Student’s *t*-test using GraphPad Prism 6.05 software. The differences between values for treated and control cells were considered significant if the *p*-value was <0.05.

### Susceptibility Testing of Planktonic Cells to Azoles and Farnesol

Antifungal susceptibility of *C. auris* isolates to fluconazole, itraconazole, voriconazole, posaconazole, isavuconazole and to farnesol (all from Sigma, Budapest, Hungary) was tested using the broth microdilution method in RPMI-1640 in line with the CLSI standard M27-A3 guideline (Clinical and Laboratory Standards Institute, 2008). The final concentrations of the drug ranged between 0.5 and 32 mg/L, 0.008 and 0.5 mg/L, and 1.17 and 300 μM mg/L for fluconazole, other tested azoles and farnesol, respectively. Susceptibility testing for planktonic cells was performed in 96-well microtiter plates at 35°C for 24 h. The inoculum was 0.5–2.5 × 10^3^ cells/mL. Minimum inhibitory concentrations (MICs) were defined as at least 50% growth reduction compared with untreated control. All isolates were tested in three independent experiments and the median of the three values was used in the analysis.

### Biofilm Formation

*Candida* isolates were suspended in RPMI-1640 broth at a concentration of 1 × 10^6^ cells/mL and aliquots of 100 μL were inoculated onto flat-bottom 96-well sterile microtiter plates (TPP, Trasadingen, Switzerland) and then incubated statically at 35°C for 24 h to produce one-day-old biofilms (Pierce et al., 2008; Kovács et al., 2016).

### Metabolic Activity Changes of Biofilms Over Time Following Farnesol Exposure

The effect of pre-exposure and continuous farnesol treatment on *C. auris* and *C. albicans* biofilms was tested in three experimental settings: (i) continuous farnesol treatment for 24-h during biofilm formation, (ii) biofilm forming ability of cells pre-exposed with farnesol (75 μM) for 24-h prior to biofilm formation then continuously treated to given farnesol concentrations for 24-h during biofilm development, (iii) effect of farnesol on one-day-old biofilms. Farnesol concentrations tested were 10, 50, 100, and 300 μM in all experiments. Metabolic activity of sessile cells was determined at 0, 2, 4, 6, 8, 10, 12, and 24 h using XTT-reduction assay (Hawser, 1996; Katragkou et al., 2015). All isolates were tested in three independent experiments and the mean of the three values was used in the analysis. At given time points, one-way ANOVA with Dunnett’s post-testing was used to analyze the metabolic activity change exerted by different farnesol concentrations compared to untreated control. The differences between values for treated and control cells were considered significant if the *p*-value was lower than 0.05.

### Susceptibility Testing of Biofilms

The activity of triazoles and farnesol against one-day-old biofilms was evaluated using the XTT-assay (Hawser, 1996; Katragkou et al., 2015; Kovács et al., 2016; Nagy et al., 2019). The concentrations tested in biofilm MIC determination ranged between 8 and 512 mg/L, 0.5 and 32 mg/L, 0.125 and 8 mg/L, and 1.17 and 300 μM for fluconazole, voriconazole/itraconazole, posaconazole/isavuconazole and farnesol, respectively. To determine the 24-h biofilm MICs, one-day-old biofilms were first washed three times with 200 μL sterile physiological saline. All wells were filled with 100 μL of 0.5 g/L XTT/1 μM menadione solution. The plates were covered and incubated at 35°C for 2 h; afterward, 80 μL of the supernatant was removed and transferred into a new sterile 96-well plate to measure the absorbance spectrophotometrically at 492 nm. MICs were defined as the lowest concentration that produced at least 50% reduction in metabolic activity of fungal biofilms compared to untreated control (Katragkou et al., 2015; Kovács et al., 2016; Nagy et al., 2019). Three independent experiments were performed for all isolates and the median of the three values were presented.

### *In vitro* Interactions Between Farnesol and Azoles for Planktonic Cells and Biofilms

A fractional inhibitory concentration index (FICI) was used to evaluate drug-drug interactions using a two-dimensional broth microdilution checkerboard assay both for planktonic and sessile cells (Meletiadis et al., 2005; Katragkou et al., 2015; Kovács et al., 2016). In the case of *C. albicans*, combinations were tested only for biofilms because planktonic isolates are generally susceptible to the tested azoles. The concentration ranges were as described above for MIC determination against planktonic cells and biofilms. The FICI expressed as ΣFIC = FIC_A_ + FIC_B_ = MIC_A_^combination^/MIC_A_^alone^ + MIC_B_^combination^/MIC_B_^alone^, where MIC_A_^alone^ and MIC_B_^alone^ are the MIC values of compounds A and B used alone and MIC_A_^combination^ and MIC_B_^combination^ are the MICs of compounds A and B at the isoeffective combinations, respectively. FICI was defined as the lowest ΣFIC (Meletiadis et al., 2005; Katragkou et al., 2015; Kovács et al., 2016). The MIC values of the drugs alone and of all isoeffective combinations were determined as the lowest drug concentrations showing at least 50% reduction of turbidity for planktonic, or at least 50% reduction in metabolic activity of biofilm compared to the untreated control cells. The interaction between azoles and farnesol was interpreted as synergistic when FICI was ≤0.5, as indifferent interaction when FICI was between >0.5 and 4 and as antagonism when FICI was >4 (Meletiadis et al., 2005; Katragkou et al., 2015; Kovács et al., 2016).

### *In vivo* Experiments

BALB/c immunocompromised female mice (21–23 g) (Charles River) were used to examine the effect of farnesol pre-exposure (75 μM) and daily farnesol treatment (75 μM) on virulence of *C. auris* and compared to *C. albicans* SC5314. The animals were maintained in accordance with the Guidelines for the Care and Use of Laboratory Animals. The experiments were approved by the Animal Care Committee of the University of Debrecen, Debrecen, Hungary (permission no. 12/2014 DEMÁB). Permanent immunosuppression was produced by intraperitoneal administration of 150 mg/kg cyclophosphamide 4 days prior to infection, 100 mg/kg cyclophosphamide 1 day prior to infection, 100 mg/kg cyclophosphamide 2 days post-infection and 100 mg/kg cyclophosphamide 5 days post-infection (Andes et al., 2010; Kovács et al., 2014). In accordance with our preliminary experiments, mice were challenged intravenously through the lateral tail vein; the infectious doses were 1 × 10^7^ CFU/mouse and 8 × 10^3^ CFU/mouse in 0.2 mL volume for *C. auris* and *C. albicans*, respectively. Inoculum density was confirmed by plating serial dilutions on Sabouraud dextrose agar (Kovács et al., 2014). Mice were divided into four groups (10 mice per group); (i) untreated control mice; (ii) inoculation with 24 h-long farnesol pre-exposed (75 μM) cells; (iii) there was no farnesol pre-exposure to fungal cells prior to infection, but 75 μM daily farnesol treatment (corresponding to approximately 0.4 mg/kg) was started from 24 h post-infection; (iv) 24 h-long farnesol pre-exposure (75 μM) to fungal cells prior to infection; afterward, 75 μM daily farnesol treatment was started at 24 h post-infection.

Farnesol treatments were administered intraperitoneally in a volume of 0.5 mL. Control mice were given 0.5 mL physiological saline intraperitoneally. At 6 days post-infection, mice were euthanized, and their kidneys were removed (Fakhim et al., 2018), weighed and homogenized aseptically. Fungal tissue burden was determined by quantitative culturing. Kidney tissue burden was analyzed using Kruskal–Wallis test with Dunn’s post-test (GraphPad Prism 6.05). Significance was defined as *p* < 0.05.

### Histology

Kidneys of treated and untreated mice were subjected to histological investigations. Histopathological examination and histochemical staining were performed on routine formalin-fixed, paraffin-embedded mouse kidney tissues. Serial 4-μm-thick sections were cut from paraffin blocks, and Periodic acid-Schiff (PAS) staining was performed (Pupim et al., 2017; Kovács et al., 2019).

## Results

### Effect of Farnesol on *C. auris* and *C. albicans* Planktonic Cell Growth

**FIGURE 1:**
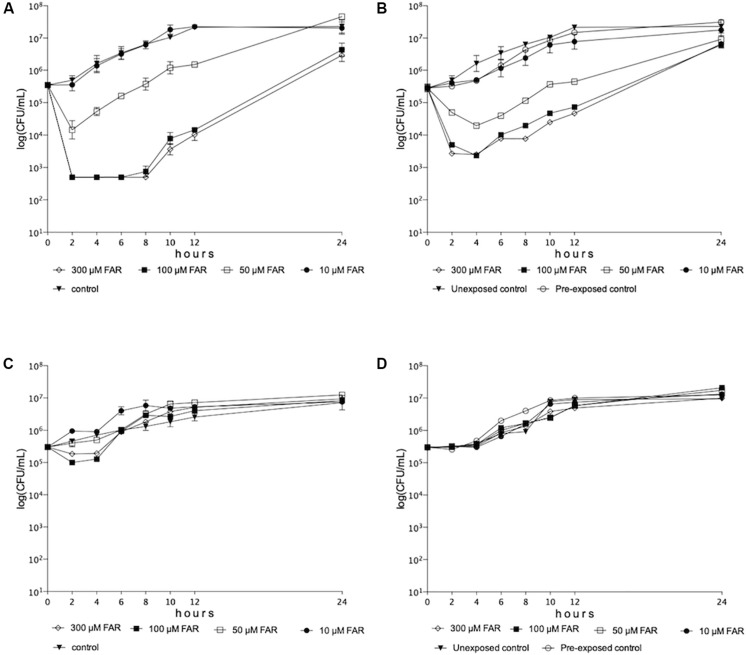
Time-kill curves of farnesol against *Candida auris* **(A,B)** and *Candida albicans* **(C,D)** isolates in RPMI-1640 for farnesol unexposed **(A,C)** and farnesol pre-exposed **(B,D)** cells (75 μM), respectively. Each timepoint represents mean ± SEM (standard error of mean) of cell count derived from isolates.

Significant decrease was observed in growth rate of *C. auris* for 12 h in the presence of farnesol concentrations ranges from 50 to 300 μM both in case of farnesol unexposed and pre-exposed cells (*p* < 0.001-0.05) (Figures 1A,B). At 24 h, 100 and 300 μM farnesol significantly decreased the viable cell count compared to untreated control in both experimental settings (*p* < 0.01–0.001) (Figures 1A,B). Surprisingly, neither farnesol pre-exposed nor unexposed *C. albicans* cells showed significant growth reduction at 24 h (*p* > 0.05) (Figures 1C,D).

### Effects of Farnesol on Extracellular Phospholipase and Proteinase Production of *C. auris* and *C. albicans*

Farnesol treatment did not significantly influence the extracellular proteinase activity of either *C. auris* or *C. albicans*. The *Pz* values were 0.83 ± 0.04 and 0.82 ± 0.05 for *C. auris* untreated control and farnesol-exposed cells, respectively (*p* > 0.05), as compared to 0.53 ± 0.003 and 0.48 ± 0.02 with *C. albicans* untreated control and farnesol-exposed cells, respectively (*p* > 0.05). Farnesol exposure resulted in significantly higher phospholipase activity for *C. albicans* (*Pz* values were 0.48 ± 0.04 and 0.42 ± 0.02 for untreated control and farnesol-exposed cells, respectively (*p* < 0.01); however, the *Pz* values were statistically comparable in case of *C. auris* (*Pz* values were 0.9 ± 0.04 and 0.89 ± 0.05 for untreated control and farnesol-exposed cells, respectively (*p* > 0.05).

### Farnesol-Induced Oxidative Stress and Stress Response in *C. auris* and *C. albicans*

Farnesol caused a significantly higher reactive species production in *C. auris* compared with untreated control cells as presented in Table 1 (*p* < 0.001). This farnesol-related higher reactive species level was associated with elevated superoxide dismutase (*p* < 0.001) but statistically comparable catalase activity (*p* > 0.05) (Table 1). Farnesol treatment did not result in significantly higher reactive species production in *C. albicans* (*p* > 0.05), which is in line with the statistically comparable catalase and superoxide dismutase activity between farnesol exposed cells and untreated control (*p* > 0.05) (Table 1).

**TABLE 1:** Farnesol-induced oxidative stress response in *Candida auris* and *Candida albicans.*

**Oxidative stress related parameter**	**Untreated cultures**		**Farnesol-exposed cultures**	
		
	***C. auris***	***C. albicans***	***C. auris***	***C. albicans***
Catalase [kat (kg protein)^–1^]	1.410.03	0.600.07	1.560.09	0.480.07
SOD [munit (mg protein)^–1^]	85.695.42	78.134.51	170.1117.37***	81.416.12
DCF [nmol DCF (OD_640_)^–1^]	3.960.89	9.691.01	23.544.51***	11.451.15

Mean ± standard deviation values calculated from three independent experiments are presented. ***Significant differences at *p* < 0.001, as calculated by the paired Student’s *t*-test compared to untreated control and farnesol-treated cultures for *C. auris*. SOD, superoxid dismutase; DCF, 2′,7′-dichlorofluorescein.

### Effects of Farnesol on Biofilm Forming Ability and One-Day-Old Biofilms of *C. auris* and *C. albicans*

#### The Effect of Different Farnesol Concentrations on Biofilm Forming Ability

**FIGURE 2:**
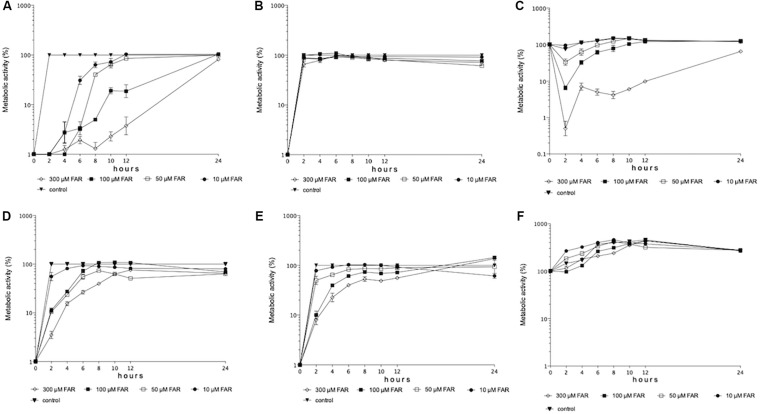
Metabolic activity changes over time in case of biofilm formation in the presence of given farnesol concentrations (10–300 μM) for *C. auris* **(A)** and *C. albicans* **(D)**, respectively. Metabolic activity changes over time in case of biofilm formation by farnesol pre-exposed cells (75 μM) in the presence of given farnesol concentrations (10–300 μM) for *C. auris* **(B)** and *C. albicans* **(E)**, respectively. Metabolic activity changes over time for one-day-old preformed biofilms in the presence of given farnesol concentrations (10–300 μM) for *C. auris* **(C)** and *C. albicans* **(F)**, respectively. Each time-point represents mean ± SEM (standard error of mean) of metabolic activity of clinical isolates (three independent experiments per isolate).

All tested farnesol concentrations inhibited the metabolic activity of *C. auris* cells compared to control cells at first 8 h (*p* < 0.001-0.05); while, statistically comparable metabolic activities were measured at 24 h (*p* > 0.05) (Figure 2A). In contrast, all tested farnesol concentrations inhibited the metabolic activity of *C. albicans* cells compared to untreated control at 24 h (Figure 2D).

#### Biofilm Forming Ability of Cells Pre-exposed With Farnesol for 24-h (75 μM) Prior to Biofilm Formation

Interestingly, we observed statistically significant differences in metabolic activity of *C. auris* cells only at 24 h between 50 and 300 μM (Figure 2B). In the case of *C. albicans*, statistically significant differences in metabolic activity between 50 and 300 μM were first observed at 8 h (Figure 2E), but the metabolic activity of cells treated by various concentrations was statistically comparable at 24 h (Figure 2E).

#### The Effect of Different Farnesol Concentrations Against One-Day-Old Biofilms

Between 2 and 24 h, 300 μM farnesol produced a potent anti-biofilm effect against *C. auris* compared to control (Figure 2C). Interestingly, the low farnesol concentrations (10–50 μM) increased the metabolic activity of *C. albicans* biofilms in the first 4 h (Figure 2F). However, the various farnesol treatments were statistically comparable against *C. albicans* at 24 h (Figure 2F).

### Susceptibility Results for Planktonic Cells and Biofilms

For *C. auris* isolates, the planktonic MICs ranged from 4 to >32 mg/L, from 0.03 to 0.06 mg/L, from 0.008 to 0.015 mg/L, from 0.015 to 0.03 mg/L, and from 0.008 to 0.015 mg/L for fluconazole, voriconazole, isavuconazole, itraconazole and posaconazole, respectively. The susceptibility to fluconazole of isolate 10 was higher than the tentative fluconazole MIC breakpoint (>32 mg/L) while the other two strains were susceptible to fluconazole (Centers for Disease Control and Prevention, 2020). In the case of planktonic *C. albicans* SC5314 reference strain, the median MIC values were 0.125 mg/L, 0.015 mg/L, 0.015 mg/L, 0.125 mg/L, and 0.008 mg/L for fluconazole, voriconazole, isavuconazole, itraconazole and posaconazole, respectively. In case of biofilms, the median MIC values are shown in Table 2.

**TABLE 2:** Minimum inhibitory concentration of fluconazole (FLU), voriconazole (VOR), itraconazole (ITRA), posaconazole (POSA) and isavuconazole (ISA) alone and in combination with farnesol (FAR) against *Candida auris* (10, 12, and 27) and *Candida albicans* SC5314 biofilms (sMIC).

**Isolates**	**Median sMIC values**				**Interaction analysis**	
		
	**sMIC alone**		**sMIC in combination**		**Median FICI**	**Type of interaction**
	**FLU (mg/L)**	**FAR (μM)**	**FLU (mg/L)**	**FAR (μM)**		
10	>512^a^	300	64	75	0.375	Synergy
12	>512^a^	300	64	75	0.35	Synergy
27	>512^a^	300	64	75	0.375	Synergy
SC5314	>512^a^	150	64	75	0.56	Indifferent

	**VOR (mg/L)**	**FAR (μM)**	**VOR (mg/L)**	**FAR (μM)**		

10	64	150	0.5	4.69	0.093	Synergy
12	64	300	0.5	4.69	0.061	Synergy
27	64	300	0.5	9.38	0.038	Synergy
SC5314	16	150	1	4.69	0.09	Synergy

	**ITRA (mg/L)**	**FAR (μM)**	**ITRA (mg/L)**	**FAR (μM)**		

10	16	300	0.5	4.69	0.155	Synergy
12	32	300	0.5	9.375	0.140	Synergy
27	16	300	0.5	9.375	0.123	Synergy
SC5314	8	150	0.5	4.69	0.187	Synergy

	**POSA (mg/L)**	**FAR (μM)**	**POSA (mg/L)**	**FAR (μM)**		

10	16	150	0.25	2.34	0.062	Synergy
12	16	150	0.25	2.34	0.062	Synergy
27	16	150	0.25	2.34	0.062	Synergy
SC5314	2	150	0.25	4.69	0.28	Synergy

	**ISA (mg/L)**	**FAR (μM)**	**ISA (mg/L)**	**FAR (μM)**		

10	4	300	0.125	9.38	0.091	Synergy
12	8	300	0.125	18.75	0.062	Synergy
27	4	300	0.125	9.38	0.091	Synergy
SC5314	8	150	0.5	4.69	0.28	Synergy

*Furthermore, *in vitro* interactions by fractional inhibitory concentration index (FICI) determination of fluconazole, voriconazole, itraconazole, posaconazole, and isavuconazole in combination with farnesol against *C. auris* and *C. albicans* biofilms. Median MIC values and FICI values from three independent experiments are presented. ^*a*^MIC is off-scale at >512 mg/l, 1024 mg/l (one dilution higher than the highest tested concentration) was used for analysis.*

### Interactions Between Triazoles and Farnesol by FICI

Only indifferent interactions were detected for planktonic cells of *C. auris* (data not shown). The results of the triazole-farnesol interaction against one-day-old biofilms based on FICI are summarized in Table 2. Antagonism was never observed. Synergy between triazoles and farnesol was observed for all three *C. auris* isolates when grown in biofilm (FICI ranges from 0.038 to 0.375) (Table 2). For the *C. albicans* SC5314 strain, the interaction pattern observed was very similar to *C. auris*; an indifferent interaction between an azole and farnesol was observed only in case of fluconazole, although, the FICI value calculated was very close to the synergy threshold (Table 2).

### *In vivo* Experiments

**FIGURE 3:**
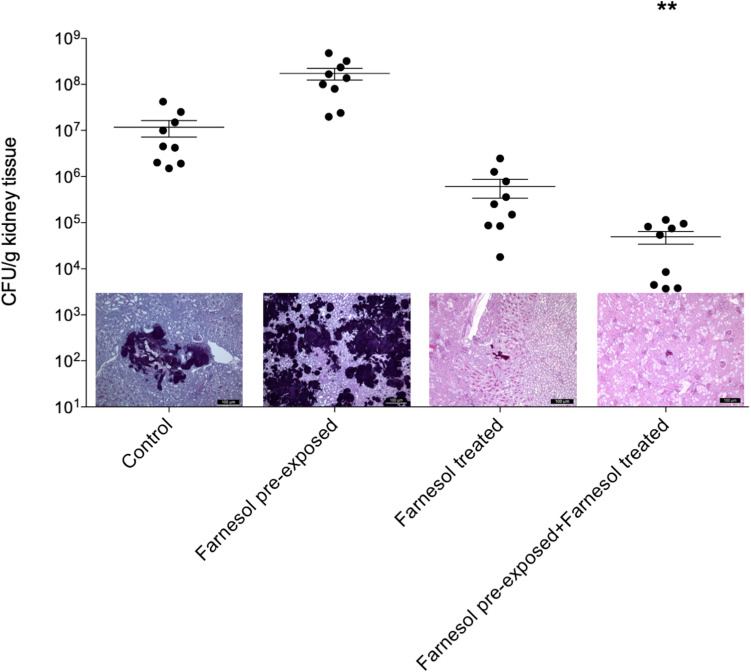
The kidney burden of *Candida auris* in a systemically infected mouse model. The bars represent the means ± SEM (standard error of mean) of kidney tissue burdens of BALB/c mice. Significant differences between CFU numbers were determined based on comparison with the untreated controls. Levels of significant differences are indicated (**p < 0.01). Histological changes in kidney tissue from mice suffering from systemic candidiasis with or without farnesol treatment in the presence or absence of farnesol pre-exposure were examined by Periodic acid-Schiff staining.

**FIGURE 4:**
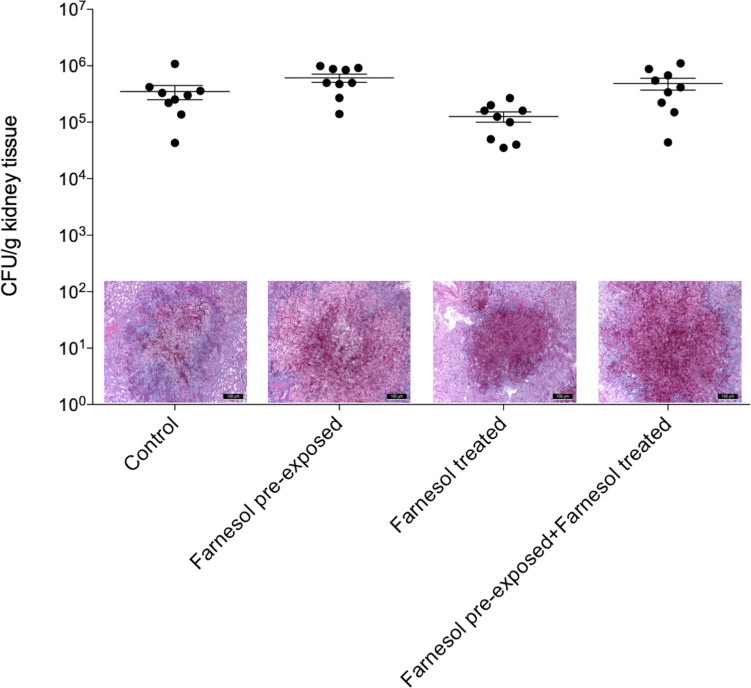
The kidney burden of *Candida albicans* in a systemically infected mouse model. The bars represent the means ± SEM (standard error of mean) of kidney tissue burdens of BALB/c mice. Significant differences between CFU numbers were determined based on comparison with the untreated controls. Histological changes in kidney tissue from mice suffering from systemic candidiasis with or without farnesol treatment in the presence or absence of farnesol pre-exposure were examined by Periodic acid-Schiff staining.

Results of the *in vivo* experiments are shown in Figures 3, 4 for *C. auris* and *C. albicans*, respectively. Seventy-five μM farnesol treatment decreased the fungal kidney burden especially when farnesol pre-exposed *C. auris* cells were used as inoculum (Figure 3). With *C. albicans*, all experimental settings resulted in statistically comparable kidney fungal burdens compared to untreated control (Figure 4). The histopathology results observed were in line with the fungal burden-related results. *C. auris* produced single yeast cells and numerous budding yeast cells in untreated control mice. Although, inoculation by farnesol pre-exposed cells caused large number of aggregates in kidney tissue; the daily farnesol treatment markedly decreased the number of lesions (Figure 3). Both farnesol pre-exposure and daily farnesol treatment caused several extended fungal lesions in kidney tissue in the case of *C. albicans* infection (Figure 4), where single and budding yeast cells, pseudohyphae and hyphae were observed in all groups (Figure 4).

## Discussion

Only a few classes of antifungal agents are available for the treatment of fungal infections; in addition, the antifungal drug discovery pipeline is slow and challenging, especially in case of the newly emerging difficult-to-treat species such as *C. auris* (Roemer and Krysan, 2014; Scorzoni et al., 2017). Combination based therapeutic approaches have been proposed as alternatives in recent years to treat the *C. auris* infections. The combination of flucytosine with amphotericin B or micafungin may be relevant for the treatment of *C. auris* infections (Bidaud et al., 2019). Moreover, synergistic interactions were observed between micafungin and voriconazole (Fakhim et al., 2017).

The investigations of alternative/adjuvant treatments focusing on fungal quorum-sensing molecules (e.g., farnesol, tyrosol) have become an intensely researched area in recent years (Mehmood et al., 2019). Several *in vitro* and *in vivo* studies were performed to evaluate the antimicrobial effects of farnesol, which revealed that this compound may potentially serve as an alternative or adjuvant drug (Jabra-Rizk et al., 2006; Hisajima et al., 2008; Katragkou et al., 2015; Bozó et al., 2016; Kovács et al., 2016; Nagy et al., 2019). Farnesol has a versatile effect at physiological concentrations, however, the most prominent of these is its ability to influence *C. albicans* morphology without markedly changing proliferation (Hornby et al., 2001). It is noteworthy that farnesol not only affects *C. albicans* but has a remarkable inhibitory effect on other non-*albicans* species and molds especially in supraphysiological concentrations (Jabra-Rizk et al., 2006; Henriques et al., 2007; Rossignol et al., 2007; Weber et al., 2010; Kovács et al., 2016). Our recent study reported that farnesol has a potential antifungal effect against *C. auris* biofilms (Nagy et al., 2019), nevertheless, the physiological processes underlying the observed antifungal activity of farnesol remain to be elucidated.

Farnesol did not affect the growth rate of planktonic *C. albicans*; but caused significant reduction in growth rate in the case of *C. auris*. Moreover, farnesol inhibited the metabolic activity of one-day-old biofilms in the first 24 h, a phenomenon clearly absent with *C. albicans*. The observed farnesol related effect in *C. albicans* is similar to those reported by Hornby et al. (2001).

Farnesol has been suggested to modulate virulence, since it was shown to affect virulence-associated phospholipase and aspartyl protease production in *C. albicans*. In this study, farnesol exposure resulted in significantly higher phospholipase activity for *C. albicans*, which is line with results reported by Fernandes et al. (2018). However, it did not enhance the production of these enzymes in experiments with *C. auris*.

Farnesol was reported to cause a dose-dependent production of reactive species and could increase resistance to oxidative stress in *C. albicans* (Davis-Hanna et al., 2008; Deveau et al., 2010), which is concordant with our results. However, farnesol treatment resulted in a significant increase of reactive species production in *C. auris*, resulting in an elevated level of superoxide dismutase but not catalase, demonstrating that farnesol might not contribute to protection against oxidative stress in *C. auris*. Such stress-related differences between *C. albicans* and *C. auris* were also observed previously with other stressor compounds. *C. auris* was more resistant to hydrogen-peroxide compared to *C. albicans*; but it was less tolerant to the superoxide-generating agent menadione and the tert-butyl hydroperoxide, and moreover displayed significantly higher resistance to cationic stress imposed by either sodium chloride or calcium chloride compared to *C. albicans* (Day et al., 2018).

To date, catheter-associated infections caused by *C. auris* have been reported by several authors, which are attributable to the previously well-documented biofilm-forming ability of this species (Dewaele et al., 2018). Previous studies reported the frequency of central line infections by *C. auris* to be between 11 and 92% (Lee et al., 2011; Schelenz et al., 2016; Taori et al., 2019). Although sessile communities show significantly higher resistance to the majority of frequently used antifungals compared to planktonic susceptibilities (Kean and Ramage, 2019), the efficacy of such antifungal agents can be enhanced using adjuvants such as farnesol (Nagy et al., 2019). A clear synergy between the tested triazoles and farnesol against *C. auris* biofilms was demonstrated, similarly to the combinations of echinocandins and farnesol (Nagy et al., 2019). Farnesol modulates the expression of genes linked to ergosterol biosynthesis, which may explain the synergy of this compound with triazoles (Yu et al., 2012).

Although the *in vitro* effect of farnesol is well known especially against *C. albicans*, its *in vivo* role remains controversial and raises several questions. Navarathna et al. (2007) showed that exogenous farnesol (20 mM/mouse) can enhance the pathogenicity of *C. albicans*, increasing the mortality in a murine model of systemic candidiasis. In contrast, Hisajima et al. (2008) observed a farnesol-induced protective effect (at a dose 9 μM/mouse) in *C. albicans*-associated oropharyngeal candidiasis. Although Bozó et al. (2016) revealed that farnesol alone is not protective in a murine vulvovaginitis model (150–300 μM/mouse), it did enhance the fluconazole activity against a fluconazole-resistant *C. albicans* isolate. In addition, chitosan nanoparticles containing miconazole and farnesol also inhibited fungal proliferation in a mouse vulvovaginitis model at ≥240 μM (Fernandes Costa et al., 2019). To the best of our knowledge, there is no reported data concerning the *in vivo* activity of farnesol against non-*albicans Candida* species. In this study, daily farnesol treatment decreased the *C. auris* fungal burden in mouse kidneys regardless of previous farnesol exposure of the inoculum. In addition, in the case of inocula pre-exposed to farnesol, the reduction of fungal cell numbers was statistically significant, which is concordant with our *in vitro* growth-related results. The antifungal activity observed may be explained by the elevated levels of reactive species previously measured *in vitro*, which could not be detected in equivalent experiments with *C. albicans*. Furthermore, the amphiphilic properties of farnesol allows for its integration into cell membranes, affecting membrane fluidity and integrity (Bringmann et al., 2000; Funari et al., 2005; Jabra-Rizk et al., 2006; Scheper et al., 2008). Farnesol was shown to affect cellular polarization and membrane permeability in *C. parapsilosis* and *Candida dubliniensis* (Jabra-Rizk et al., 2006; Rossignol et al., 2007), which may also explain the observed antifungal effect in our study. However, it is noteworthy that the inoculation of farnesol pre-exposed cells without daily farnesol treatment resulted in a more virulent *C. auris* population and increased fungal burden. The 24-hours-long pre-exposure without further continuous treatment of farnesol may influence the expression of virulence determinants or membrane properties similar to fluconazole pre-treatment, which may explain the virulence enhancer effect reported previously (Navarathna et al., 2005).

## Conclusion

In conclusion, our results clearly demonstrate farnesol-related differences in physiology between *C. albicans* and *C. auris*. Based on our *in vivo* studies, farnesol has a remarkable therapeutic potential against *C. auris*; in addition, it reverses the well-documented resistance to newer triazoles reported for *C. auris* biofilms. However, further genome-wide gene expression analysis with *C. auris* is needed in order that each aspect of farnesol-related effects (e.g., short-term exposure vs. long-term exposure) can be elucidated.

## Data Availability Statement

All datasets generated for this study are included in the article/supplementary material.

## Ethics Statement

The animal study was reviewed and approved by the Animal Care Committee of the University of Debrecen, Debrecen, Hungary (permission no. 12/2014 DEMÁB).

## Author Contributions

RK conceived the ideas, analyzed the data, and wrote the manuscript. FN performed the biofilm forming related tests and susceptibility tests, *in vivo* tests, and wrote the manuscript. EV performed the growth related experiments. ÁJ performed the oxidative stress related experiments. AB provided the strains and interpreted the several results. LF performed the histological examinations. ZT performed the biofilm forming related tests, susceptibility tests, and *in vivo* tests. LM performed the *in vivo* experiments and analyzed the data.

## Conflict of Interest

LM received conference travel grants from Cidara, MSD, Astellas and Pfizer. The remaining authors declare that the research was conducted in the absence of any commercial or financial relationships that could be construed as a potential conflict of interest.
